# Severe pediatric burn injuries following a social media trend involving a microwaved toy: A case report and warning

**DOI:** 10.1016/j.jpra.2026.02.017

**Published:** 2026-03-26

**Authors:** Rory Bonner, Kirsty JG Munro

**Affiliations:** a Plastic Surgery and Burns, Ninewells Hospital, Dundee DD2 1SG, Scotland; b University of Dundee, Ninewells Hospital & Medical School, DD1 9SY, Scotland

**Keywords:** Facial burn, Pediatric burn, Social media, Safeguarding

## Abstract

We present a case report of a 10-year-old girl who sustained significant facial burns after microwaving a frozen stress ball; just one example of a “trend” which quickly gained popularity without disclaiming the harmful risks. The management and outcome of a significant contact burn linked to an uncensored online activity is described, highlighting important considerations of facial burn management and discussing the broader implications for pediatric safety in the ever-evolving digital age.

## Introduction

### Background/rationale

Social media platforms have increasingly become sources of behavioral influence. We present a case report of a 10-year-old girl who sustained significant facial burns after microwaving a frozen stress ball; just one example of a “trend” which quickly gained popularity without disclaiming the harmful risks. The younger demographic are particularly at risk as many are unaware of the associated dangers of these “challenges” leading to burn injuries or other serious, possibly fatal, sequelae, such as: consuming unsafe amounts of antihistamines (“Benadryl Challenge”); inhaling toxic fumes (“Dusting Challenge”); or choking themselves (“Blackout Challenge”) to name a few. It is likely these injuries are under-reported both by patients, given the perceived shame or guilt of hurting oneself from a trend, and clinicians, who may not appreciate just how dangerous these behaviors can be.

These trends pose a global safety concern. In many cultures the face tends to be uncovered and thus exposed to harmful substances. The face is therefore at increased risk of burn injuries which can result in significant functional, cosmetic, and psychosocial outcomes. More needs to be done to prevent them occurring.

A recent scoping review^1^ demonstrates increasingly reported injuries from social media-promoted behaviors (54 articles overall since 2010). This appears to be one of the first published case reports in the United Kingdom (UK) of its kind. May it serve as evidence to strengthen the need for better child safeguarding on social media platforms.

### Objectives

To describe the management and outcome of a significant contact burn linked to an uncensored online activity, highlighting important considerations of facial burn management and discussing the broader implications for pediatric safety in the ever-evolving digital age.

### Methods

#### Study design

This is a single case report adhering to the STROBE statement^2^ *(Supplementary Figures).*

#### Setting

Ninewells Hospital Plastic Surgery and Burns department - a burns unit in Dundee, Scotland. Laser doppler imaging is not available in the region.

#### Participants

A 10-year-old girl with no co-morbidities.

#### Variables

This study will report key interventions, assessment of burn depth, and time to healing.

#### Data sources/measurement

The local electronic patient record system was used to document all interactions with the patient and guardians, including: examination findings, photography, microbiology, and treatment plans.

Patient and guardian written informed consent was obtained for image publication.

#### Bias

Potential bias arises from the subjectivity of burn depth assessment.

#### Study size

One pediatric patient.

#### Quantitative variables

This is a descriptive study with mostly qualitative data. Time to healing is reported quantitively.

#### Statistical methods

None.

## Results

### Case description

A healthy 10-year-old female recreated a social media trend involving microwaving a frozen stress ball. The ball (available from popular UK outlets), filled with a gel-like substance, ruptured causing the hot contents to splash and stick onto the child’s face, neck, chest, and left hand. The patient’s mother attended to her quickly, carried out first aid by cooling with running water for 20–30 minutes, and attended the emergency department.

**Figure 1:**
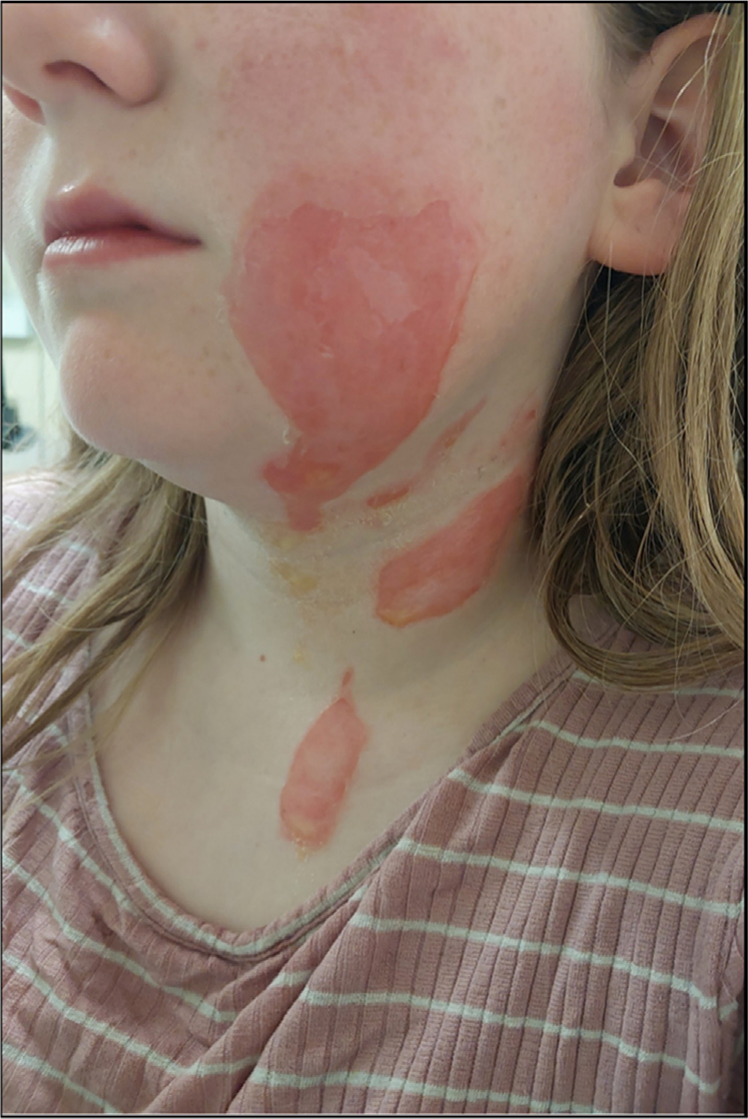
Clinical photography of burn injuries day of injury (post-debridement).

The burn injuries were assessed by the plastic surgery team, debrided, swabbed for microbiology, and clinical photography was completed (Figure 1). The burns to the left cheek and left hand were deemed superficial partial thickness and those to neck were mid to deep dermal. Overall, the total body surface area affected was 1.5%. Chloramphenicol 1% ointment was applied to the face and UrgoTul Ag dressings were applied to all other areas. The patient returned within 24 h due to increased swelling *(Supplementary Figures)*. The facial burn was noted to be deeper with a more sluggish capillary refill time compared to initial assessment. As standard, we review all burn wounds at 48 h from initial assessment *(Supplementary Figures)*.

**Figure 2:**
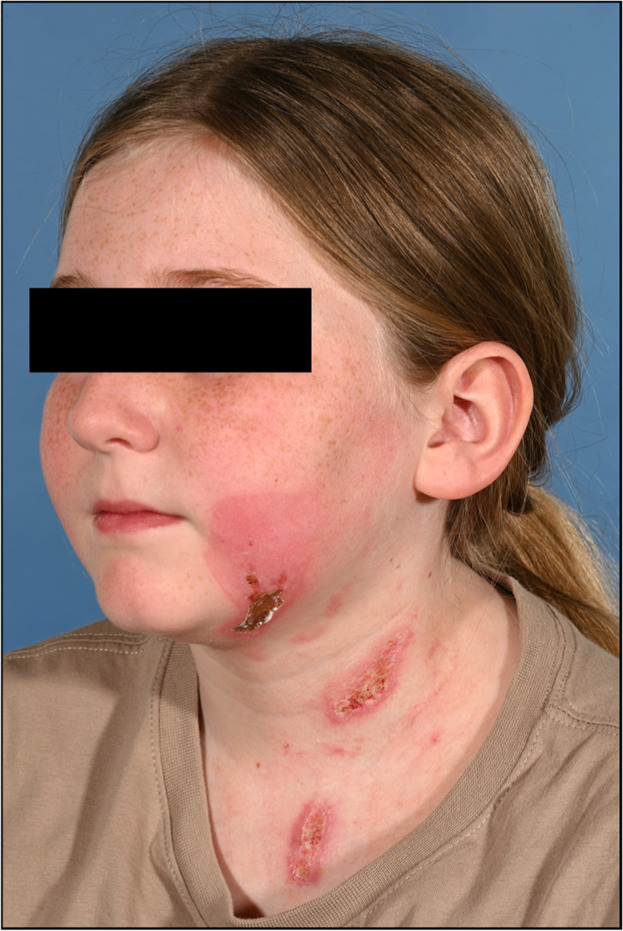
Clinical photography of burn injuries day 14 post-injury.

Weekly reviews were arranged with standard burn care well tolerated by the child in the outpatient setting. During the first two weeks (Figure 2; *and Supplementary Figures*), the area of burn lateral to the left oral commissure was non-sensate and non-blanching, surrounded by slough – we opted to dress with Flaminal® Hydro. The neck and chest demonstrated improved perfusion within the first week however were slow to re-epithelialize. Neither face nor neck/chest burns were healed within 2 weeks.

**Figure 3:**
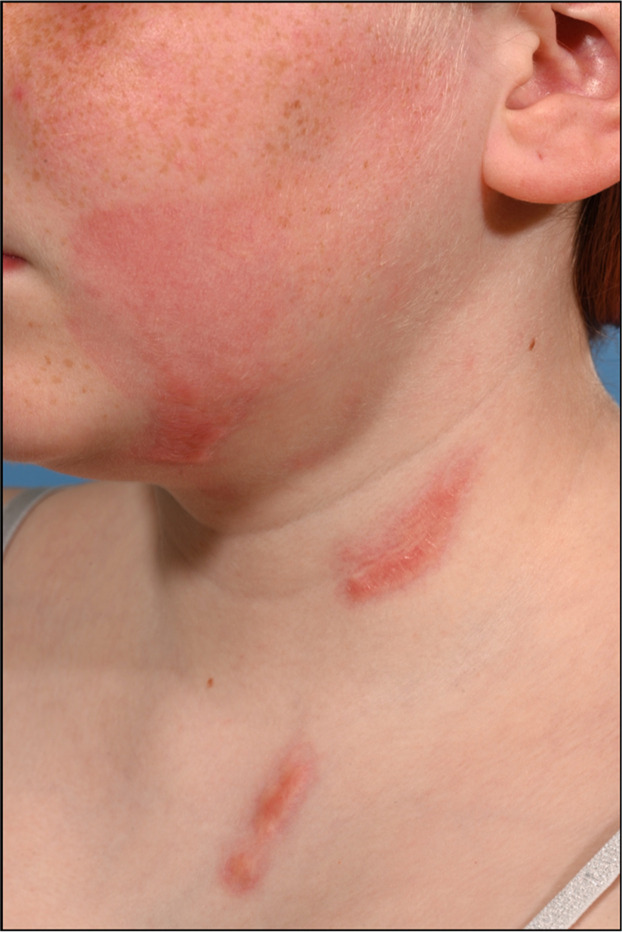
Clinical photography of burn injuries day 49 post-injury.

In-depth discussions and counselling took place between specialists, parents, and the child regarding the role of excision and skin graft reconstruction in burns not healed at 2 weeks. Both child and parent wanted to continue with non-operative intervention. The facial and hand burns were fully healed by 24 days *(Supplementary Figures)* and the neck/chest burns were healed by 49 days (Figure 3; *and Supplementary Figures*).

**Figure 4:**
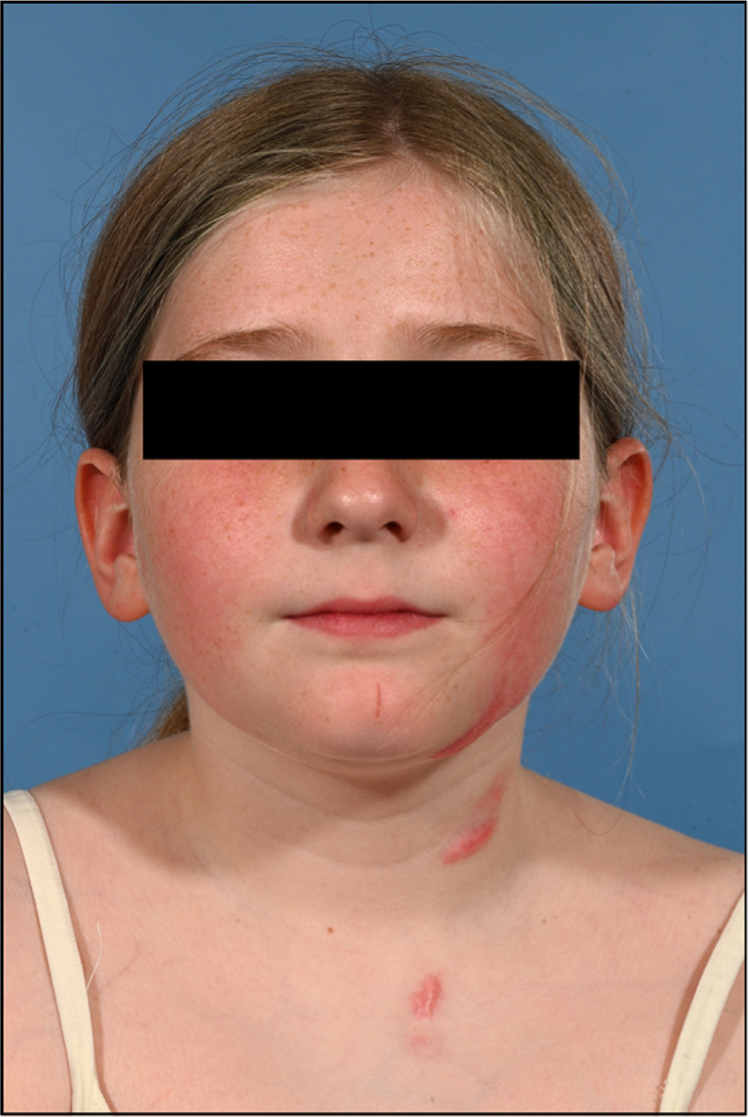
Clinical photography of burn injuries day 154 post-injury.

Unfortunately, the scars to the lower cheek, neck, and chest have become hypertrophic (Figure 4; *and Supplementary Figures*). The child remains under the scar clinic who are prescribing topical silicone gels and tape, with good effect to the neck area.

## Discussion

### Key results

This case illustrates the serious risk of seemingly innocent internet trends; particularly those involving thermal and chemical components. This case, amongst the first to be published on increasingly occurring injuries, reinforces the absolute need for adult supervision and digital literacy when using social media. News outlets have reported that the toy company and social media platforms have worked to remove dangerous videos.^3^ Promisingly, some provide artificial intelligence-generated warning information depending on your search terms.^4^ We implore social media platforms to address these dangerous trends earlier, preventing such significant trauma to children and other vulnerable people. Regulations within the UK, such as the Online Safety Act,^5^ are also evolving to increase child protection; the focus recently to reduce self-harm content.

The Care of Burns in Scotland (COBIS) Network have previously run campaigns about firework and hot water bottle safety.^6^ To date, we are not aware of an NHS social media initiative addressing harmful online trends. We recommend NHS health boards use their social media outlets to help prevent associated injuries; this can be done proactively with generalized safety content, or reactively when a new trend emerges. Strategies could be implemented to add warnings and disrupt the unfiltered, dangerous content. Clinicians assessing these new injuries should also escalate it to their public health and relations teams to urgently spread warnings against said trends. Collaboration with charities, societies, and government would likely improve the outreach and impact of such an initiative.

As noted, such injuries are likely underreported, limiting our ability to quantify the true impact of digital content on physical health. To our knowledge, there is currently no standard method within emergency department coding systems to capture socio-technical causes such as social media-driven behaviors. Whilst ICD-11 recognizes social media use disorder (SMUD), this does not extend to physical injuries instigated by digital content. In the interim, input of a standardized descriptor (such as “internet-related activity”) to existing narrative fields could enable future national case identification and inform targeted prevention strategies. The COBIS Network also aims to maintain a database of burn injuries in Scotland and these injuries should be captured nationally.^6^ Initial first aid and timing of surgical interventions are key in facial burns; the preservation of normal features is the overarching goal of treatment. Neutralization of the source is always the first step. The contents of similar toys are often unknown; the manufacturer of this particular toy claims it is filled with a polyvinyl alcohol (PVA) glue compound – unknown to the attending clinicians at the time. When heated, both compounds will stick to skin causing extended contact deepening any burn injury. Furthermore, they may contain unknown chemicals and should therefore be treated like a chemical burn, requiring pH measurements and at least 20 minutes of irrigation. Some solutions may also not be water-soluble and clinicians should ensure correct diluent selection. This is a key learning point from this case. Deciding when to offer surgical management, whether it’s early debridement or delayed excision and reconstruction, can be challenging. Rushing a decision may risk unnecessary procedures, whilst delay may lead to contractures and growth implications with significant functional defects.^7^^,^^8^ Early debridement of deeper dermal burns has shown improved healing and scarring outcomes.^9^ In retrospect, whilst evidence in smaller surface area burns is limited, this patient may have benefited from a more aggressive initial approach with non-excisional debridement of the injuries. This statement stands particularly true now we recognize the glue-like properties of the causative agent. Our threshold for said initial operative management is now much lower for similar mechanisms and injuries; although this patient's preference for non-surgical treatment was respected.

The next decision time was at the 2–3 week mark. Unhealed burns at this stage reaffirms significant dermal loss and have an elevated risk of scarring.^8^^,^^10^ The child and parent were counselled extensively regarding this and the likely outcomes of non-operative management versus excision and skin grafting; they opted for dressings only. At presentation, initial prognosis was favourable based on the burn’s anatomical location.^7^^,^^8^ The cheek is well-padded and vascular, dissipating the injury, lessening the burn, and increasing the chance of healing by secondary intention.^3^ Despite this premise, the burns did not heal by 2–3 weeks and progressed to hypertrophic scars following non-operative treatment. Ongoing monitoring is essential in this case as a scar contracture could distort the oral commissure. The chest, as shown, has a high risk of overactive scarring and requires the same due diligence.^11^ Despite the hypertrophic scarring, the child is not currently psychosocially affected by the appearance and has expressed ongoing preference to avoid surgery. The scarring is improving with the aforementioned scar management.

### Limitations

Burns depth assessment may differ from clinician to clinician – particularly at different stages of the healing process. This case demonstrates the challenges of accurate burn assessment and the importance of continuation of care.

### Interpretation

It is likely emergency services will be increasingly burdened by social media-related injuries. Clinicians rightly promote healthy lifestyle choices throughout medical specialties, such as: smoking cessation and reducing alcohol use; exercise and diet; and national screening adherence. What role should clinicians have in addressing online safety for children and vulnerable groups?

### Generalizability

The case highlights broader concerns of social media use and possible pediatric burn injuries which are applicable to the wider public, social media platforms, and lawmakers across the world.

Key learning points for all clinicians managing pediatric facial burns are presented.

## Funding

NHS Tayside Charitable Foundation (Ref: 568-ITAY0038).

## Declaration of competing interest

None.

## Appendix Supplementary materials

Image, application 1

Image, application 1
