# Supplementary material for: Severe pediatric burn injuries following a social media trend involving a microwaved toy: A case report and warning
Source: JPRAS Open. 2026 Mar 26;50:84–90. doi: 10.1016/j.jpra.2026.02.017 (PMC13147427; doi:10.1016/j.jpra.2026.02.017)
Supplement: Supplementary file 1 [file mmc1.docx]

STROBE Statement—checklist of items that should be included in reports of observational studies

|  | Item No. | Recommendation |  | Relevant text from manuscript |
| --- | --- | --- | --- | --- |
| **Title and abstract** | 1 | (*a*) Indicate the study’s design with a commonly used term in the title or the abstract |  | a case report |
|  |  | (*b*) Provide in the abstract an informative and balanced summary of what was done and what was found |  | We present a case report of a 10-year-old girl who sustained significant facial burns after microwaving a frozen stress ball; just one example of a “trend” which quickly gained popularity without disclaiming the harmful risks. The management and outcome of a significant contact burn linked to an uncensored online activity is described, highlighting important considerations of facial burn management and discussing the broader implications for paediatric safety in the ever-evolving digital age. |
| Introduction | | | |  |
| Background/rationale | 2 | Explain the scientific background and rationale for the investigation being reported |  | Social media platforms have increasingly become sources of behavioural influence. We present a case report of a 10-year-old girl who sustained significant facial burns after microwaving a frozen stress ball; just one example of a “trend” which quickly gained popularity without disclaiming the harmful risks. The younger demographic are particularly at risk as many are unaware of the associated dangers of these “challenges” leading to burn injuries or other serious, possibly fatal, sequelae…  It is likely these injuries are under-reported both by patients, given the perceived shame or guilt of hurting oneself from a trend, and clinicians, who may not appreciate just how dangerous these behaviours can be.  These trends pose a global safety concern…  A recent scoping review, currently in early access, demonstrates increasingly reported injuries from social media-promoted behaviours (54 articles overall since 2010). This appears to be one of the first published case reports in the United Kingdom (UK) of its kind. May it serve as evidence to strengthen the need for better child safeguarding on social media platforms. |
| Objectives | 3 | State specific objectives, including any prespecified hypotheses |  | To describe the management and outcome of a significant contact burn linked to an uncensored online activity, highlighting important considerations of facial burn management and discussing the broader implications for paediatric safety in the ever-evolving digital age. |
| Methods | | | |  |
| Study design | 4 | Present key elements of study design early in the paper |  | This is a single case report adhering to the STROBE statement |
| Setting | 5 | Describe the setting, locations, and relevant dates, including periods of recruitment, exposure, follow-up, and data collection |  | Ninewells Hospital Plastic Surgery and Burns department - a burns unit in Dundee, Scotland. |
| Participants | 6 | (*a*) *Cohort study*—Give the eligibility criteria, and the sources and methods of selection of participants. Describe methods of follow-up  *Case-control study*—Give the eligibility criteria, and the sources and methods of case ascertainment and control selection. Give the rationale for the choice of cases and controls  *Cross-sectional study*—Give the eligibility criteria, and the sources and methods of selection of participants |  | A 10-year-old girl with no co-morbidities. |
|  |  | (*b*) *Cohort study*—For matched studies, give matching criteria and number of exposed and unexposed  *Case-control study*—For matched studies, give matching criteria and the number of controls per case |  | n/a |
| Variables | 7 | Clearly define all outcomes, exposures, predictors, potential confounders, and effect modifiers. Give diagnostic criteria, if applicable |  | This study will report key interventions, assessment of burn depth, and time to healing. |
| Data sources/ measurement | 8* | For each variable of interest, give sources of data and details of methods of assessment (measurement). Describe comparability of assessment methods if there is more than one group |  | The local electronic patient record system was used to document all interactions with the patient and guardians, including: examination findings, photography, microbiology, and treatment plans.  Patient and guardian written informed consent was obtained for image publication. |
| Bias | 9 | Describe any efforts to address potential sources of bias |  | Potential bias arises from the subjectivity of burn depth assessment. |
| Study size | 10 | Explain how the study size was arrived at |  | One paediatric patient. |

Continued on next page

| Quantitative variables | 11 | Explain how quantitative variables were handled in the analyses. If applicable, describe which groupings were chosen and why |  | This is a descriptive study with mostly qualitative data. Time to healing is reported quantitively. |
| --- | --- | --- | --- | --- |
| Statistical methods | 12 | (*a*) Describe all statistical methods, including those used to control for confounding |  | n/a |
|  |  | (*b*) Describe any methods used to examine subgroups and interactions |  | n/a |
|  |  | (*c*) Explain how missing data were addressed |  | n/a |
|  |  | (*d*) *Cohort study*—If applicable, explain how loss to follow-up was addressed  *Case-control study*—If applicable, explain how matching of cases and controls was addressed  *Cross-sectional study*—If applicable, describe analytical methods taking account of sampling strategy |  | n/a |
|  |  | (*e*) Describe any sensitivity analyses |  | n/a |
| Results | | | | |
| Participants | 13* | (a) Report numbers of individuals at each stage of study—eg numbers potentially eligible, examined for eligibility, confirmed eligible, included in the study, completing follow-up, and analysed | *The content of the results section is the case description:*  A healthy 10-year-old female recreated a social media trend involving microwaving a frozen stress ball. The ball (available from popular UK outlets), filled with a gel-like substance, ruptured causing the hot contents to splash and stick onto the child’s face, neck, chest, and left hand. The patient’s mother attended to her quickly, cooled with running water for 20 to 30 minutes, and attended the emergency department.  The burn injuries were assessed by the plastic surgery team, debrided, swabbed, and clinical photography was completed (Figure 1). The burns to the left cheek and left hand were deemed superficial partial thickness and those to neck were mid to deep dermal. Overall, the total body surface area affected was 1.5%. Chloramphenicol 1% ointment was applied to the face and UrgoTul Ag dressings were applied to all other areas. The patient returned within 24 hours due to increased swelling (Supplementary Figures). The facial burn was noted to be deeper with a more sluggish capillary refill time compared to initial assessment. As standard, we review all burn wounds at 48 hours from initial assessment (Supplementary Figures).  Figure 1: Clinical photography of burn injuries day of injury.  Weekly reviews were arranged with standard burn care well tolerated by the child in the outpatient setting. During the first two weeks (Figure 2; and Supplementary Figures), the area of burn lateral to the left oral commissure was non-sensate and non-blanching, surrounded by slough – we opted for Flaminal® Hydro. The neck and chest demonstrated improved perfusion within the first week however were slow to re-epithelialise. Neither face nor neck/chest burns were healed within 2 weeks.  Figure 2: Clinical photography of burn injuries day 14 post-injury.  In-depth discussions and counselling took place between specialists, parents, and the child regarding the role of excision and skin graft reconstruction in burns not healed at 2 weeks. Both child and parent wanted to continue with non-operative intervention. The facial and hand burns were fully healed by 24 days (Supplementary Figures) and the neck/chest burns were healed by 49 days (Figure 3; and Supplementary Figures).  Figure 3: Clinical photography of burn injuries day 49 post-injury.  Unfortunately, the scars have become hypertrophic (Figure 4; and Supplementary Figures). The child remains under the scar clinic who are prescribing topical silicone gels and tape.  Figure 4: Clinical photography of burn injuries day 154 post-injury. | |
|  |  | (b) Give reasons for non-participation at each stage |  |  |
|  |  | (c) Consider use of a flow diagram |  |  |
| Descriptive data | 14* | (a) Give characteristics of study participants (eg demographic, clinical, social) and information on exposures and potential confounders |  |  |
|  |  | (b) Indicate number of participants with missing data for each variable of interest |  |  |
|  |  | (c) *Cohort study*—Summarise follow-up time (eg, average and total amount) |  |  |
| Outcome data | 15* | *Cohort study*—Report numbers of outcome events or summary measures over time |  |  |
|  |  | *Case-control study—*Report numbers in each exposure category, or summary measures of exposure |  |  |
|  |  | *Cross-sectional study—*Report numbers of outcome events or summary measures |  |  |
| Main results | 16 | (*a*) Give unadjusted estimates and, if applicable, confounder-adjusted estimates and their precision (eg, 95% confidence interval). Make clear which confounders were adjusted for and why they were included |  |  |
|  |  | (*b*) Report category boundaries when continuous variables were categorized |  |  |
|  |  | (*c*) If relevant, consider translating estimates of relative risk into absolute risk for a meaningful time period |  |  |

Continued on next page

| Other analyses | 17 | Report other analyses done—eg analyses of subgroups and interactions, and sensitivity analyses |  | n/a |
| --- | --- | --- | --- | --- |
| Discussion | | | | |
| Key results | 18 | Summarise key results with reference to study objectives |  | This case illustrates the serious risk of seemingly innocent internet trends; particularly those involving thermal and chemical components. This case, amongst the first to be published on increasingly occurring injuries, reinforces the absolute need for adult supervision and digital literacy when using social media. … We implore social media platforms to address these dangerous trends earlier, preventing such significant trauma to children and other vulnerable people. …  To date, we are not aware of an NHS social media initiative addressing harmful online trends. We recommend NHS health boards use their social media outlets to help prevent associated injuries… Strategies could be implemented to add warnings and disrupt the unfiltered, dangerous content. Clinicians assessing these new injuries should also escalate it to their public health and relations teams to urgently spread warnings against said trends. Collaboration with charities, societies, and government would likely improve the outreach and impact of such an initiative.  As noted, such injuries are likely underreported, limiting our ability to quantify the true impact of digital content on physical health. To our knowledge, there is currently no standard method within emergency department coding systems to capture socio-technical causes such as social media-driven behaviours. … In the interim, input of a standardised descriptor (such as “internet-related activity”) to existing narrative fields could enable future national case identification and inform targeted prevention strategies. The COBIS Network also aims to maintain a database of burn injuries in Scotland and these injuries should be captured nationally.  Initial first aid and timing of surgical interventions are key in facial burns… The contents of similar toys are often unknown… they may contain unknown chemicals and should therefore be treated like a chemical burn, requiring pH measurements and at least 20 minutes of irrigation. Some solutions may also not be water-soluble and clinicians should ensure correct diluent selection. This is a key learning point from this case. Deciding when to offer surgical management, whether it’s early debridement or delayed excision and reconstruction, can be challenging. ... In retrospect, whilst evidence in smaller surface area burns is limited, this patient may have benefited from a more aggressive initial approach with non-excisional debridement of the injuries. This statement stands particularly true now we recognise the glue-like properties of the causative agent. Our threshold for initial operative management is now much lower for similar mechanisms and injuries.  The next decision time was at the 2-3 week mark. Unhealed burns at this stage reaffirms significant dermal loss and have an elevated risk of scarring. … Ongoing monitoring is essential in this case as a scar contracture could pull and distort the oral commissure. The chest, as shown, has a high risk of overactive scarring and requires the same due diligence. Despite the hypertrophic scarring, the child is not currently psychosocially affected by the appearance and has expressed ongoing preference to avoid surgery. |
| Limitations | 19 | Discuss limitations of the study, taking into account sources of potential bias or imprecision. Discuss both direction and magnitude of any potential bias |  | Burns depth assessment may differ from clinician to clinician – particularly at different stages of the healing process. This case demonstrates the challenges of accurate burn assessment and the importance of continuation of care. |
| Interpretation | 20 | Give a cautious overall interpretation of results considering objectives, limitations, multiplicity of analyses, results from similar studies, and other relevant evidence |  | It is likely emergency services will be increasingly burdened by social media-related injuries. Clinicians rightly promote healthy lifestyle choices throughout medical specialties, such as: smoking cessation and reducing alcohol use; exercise and diet; and national screening adherence. What role should clinicians have in addressing online safety for children and vulnerable groups? |
| Generalisability | 21 | Discuss the generalisability (external validity) of the study results |  | The case highlights broader concerns of social media use and possible paediatric burn injuries which are applicable to the wider public, social media platforms, and lawmakers across the world.  Key learning points for all clinicians managing paediatric facial burns are presented. |
| Other information | |  | | |
| Funding | 22 | Give the source of funding and the role of the funders for the present study and, if applicable, for the original study on which the present article is based |  | NHS Tayside Charitable Foundation. |

*Give information separately for cases and controls in case-control studies and, if applicable, for exposed and unexposed groups in cohort and cross-sectional studies.

**Note:** An Explanation and Elaboration article discusses each checklist item and gives methodological background and published examples of transparent reporting. The STROBE checklist is best used in conjunction with this article (freely available on the Web sites of PLoS Medicine at http://www.plosmedicine.org/, Annals of Internal Medicine at http://www.annals.org/, and Epidemiology at http://www.epidem.com/). Information on the STROBE Initiative is available at www.strobe-statement.org.
