# Supplementary material for: Severe pediatric burn injuries following a social media trend involving a microwaved toy: A case report and warning
Source: JPRAS Open. 2026 Mar 26;50:84–90. doi: 10.1016/j.jpra.2026.02.017 (PMC13147427; doi:10.1016/j.jpra.2026.02.017)

# Supplementary Figures

## Clinical photography of burn injuries day 1 post-injury.


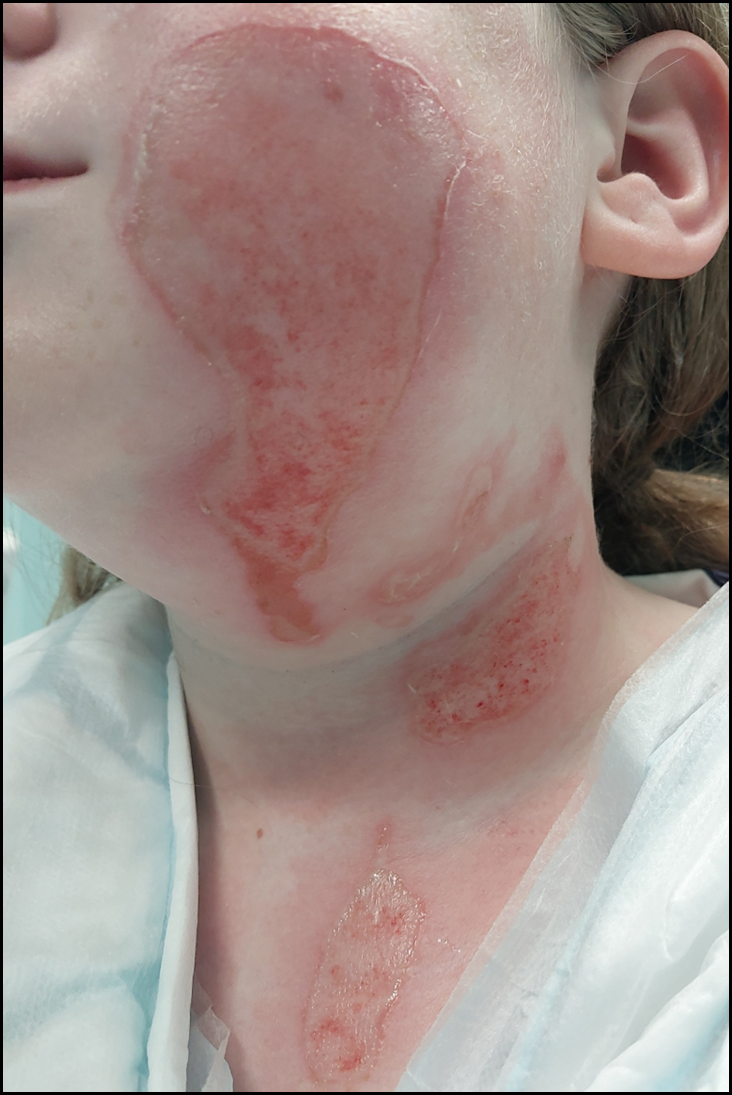


## Clinical photography of burn injuries day 3 post-injury.


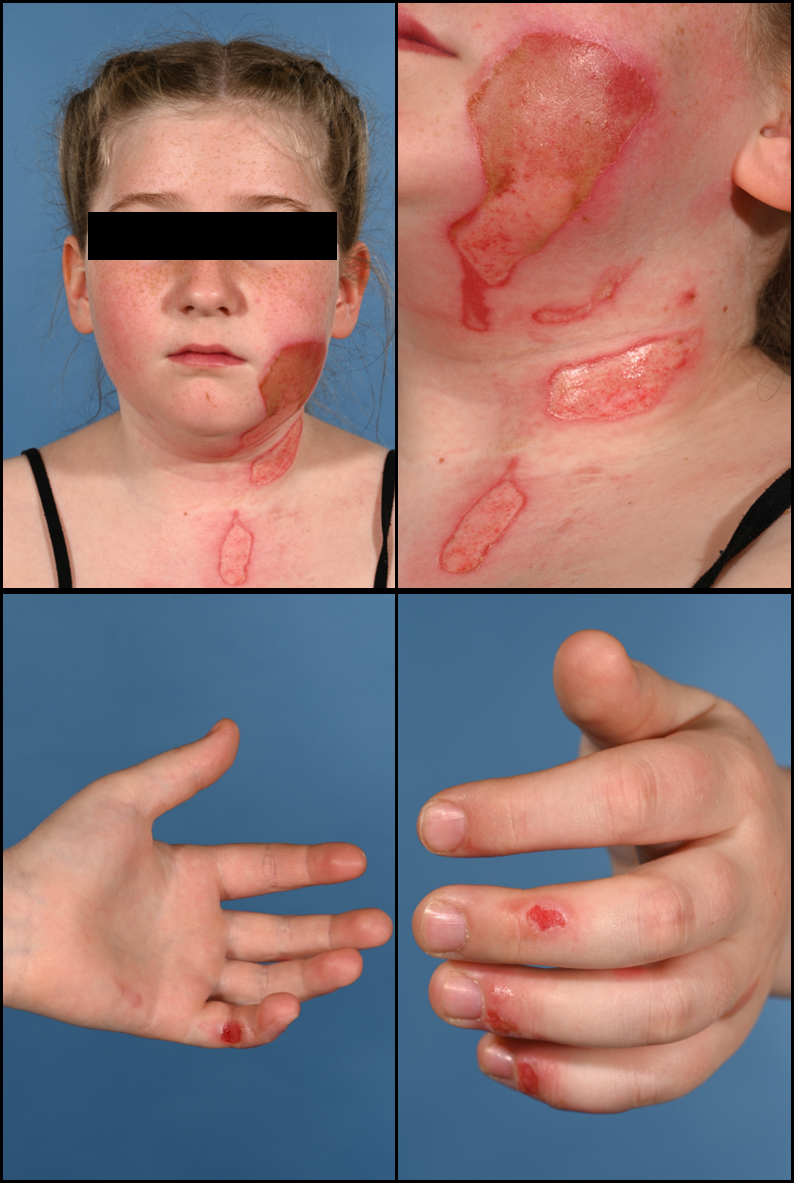


## Clinical photography of burn injuries day 14 post-injury.


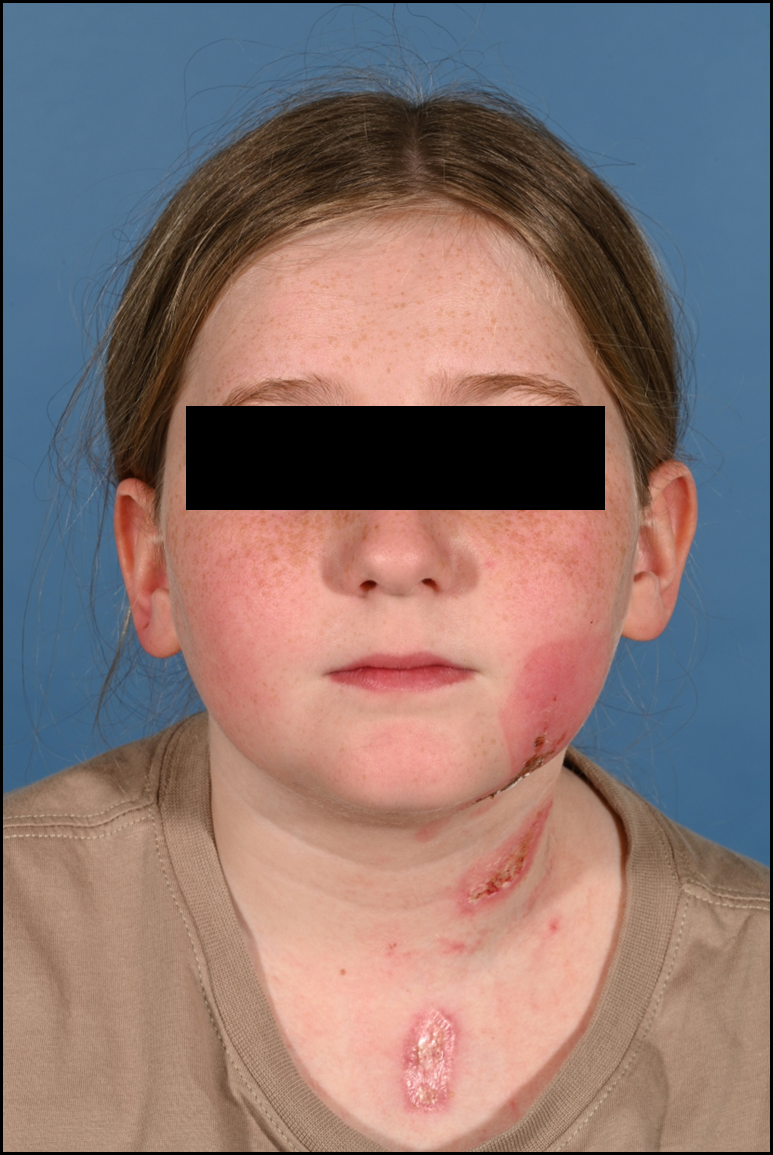


## Clinical photography of burn injuries day 24 post-injury.


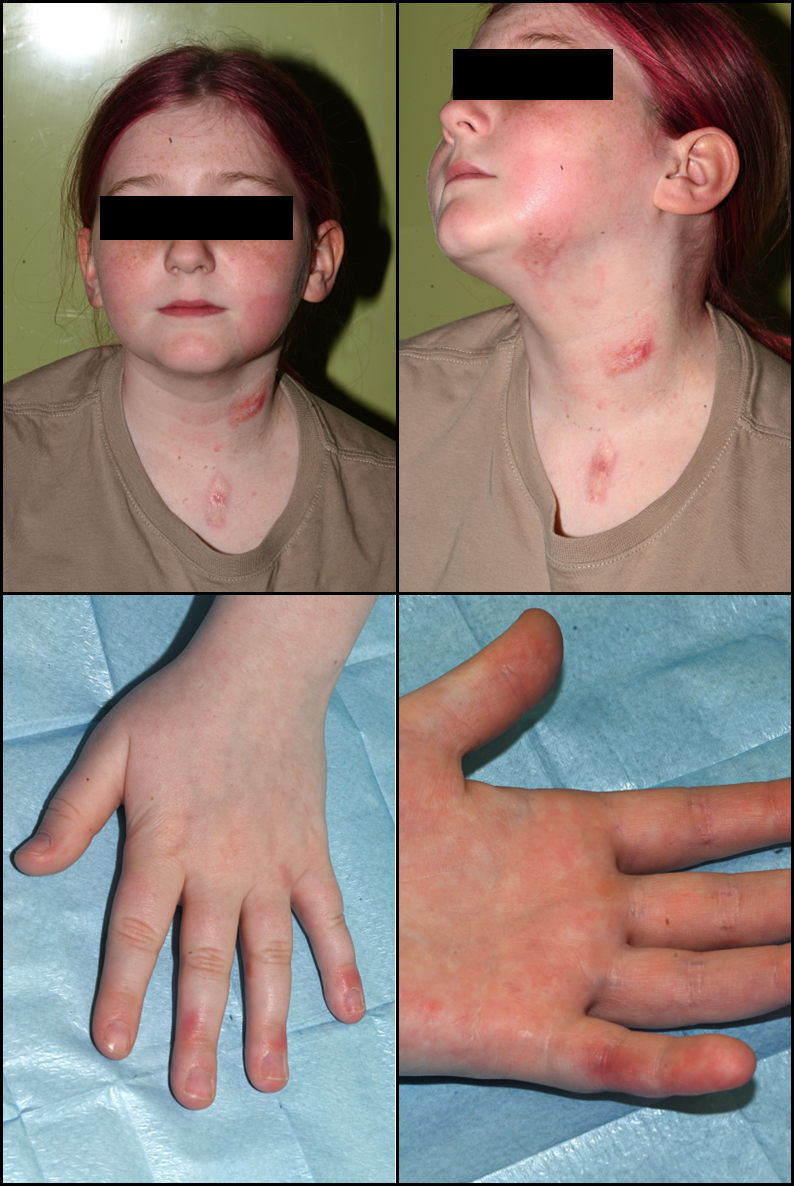


## Clinical photography of burn injuries day 49 post-injury.


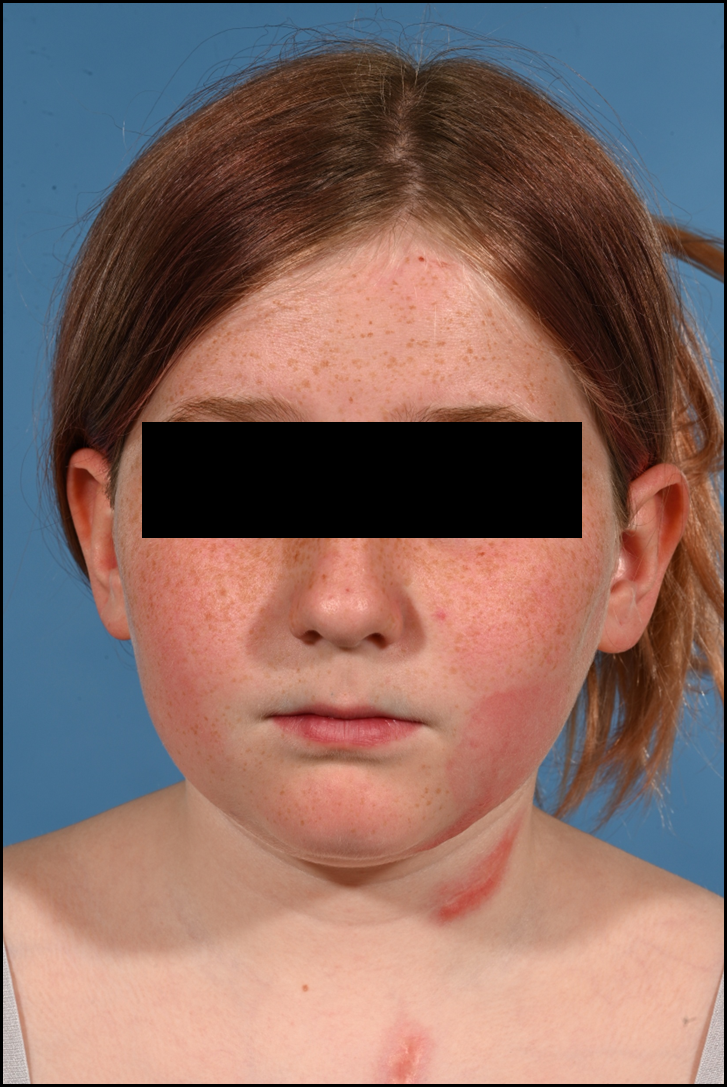


## Clinical photography of burn injuries day 154 post-injury.


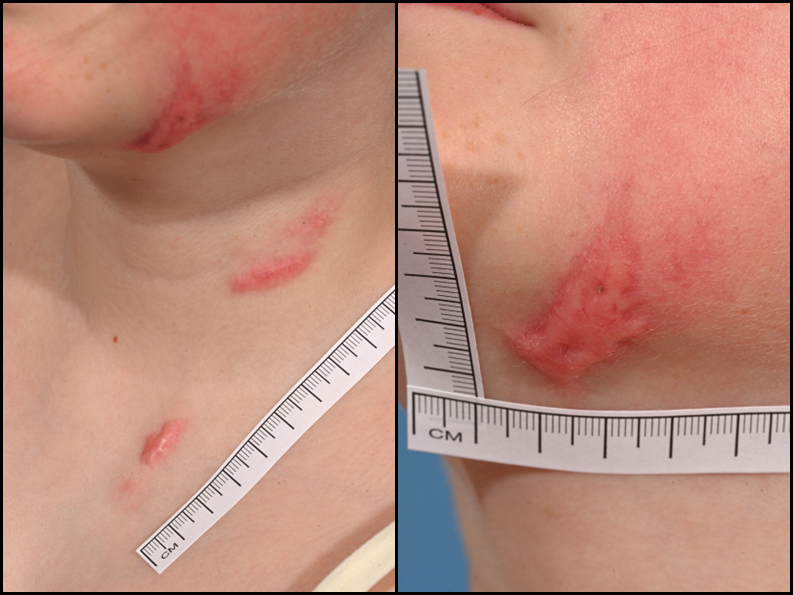

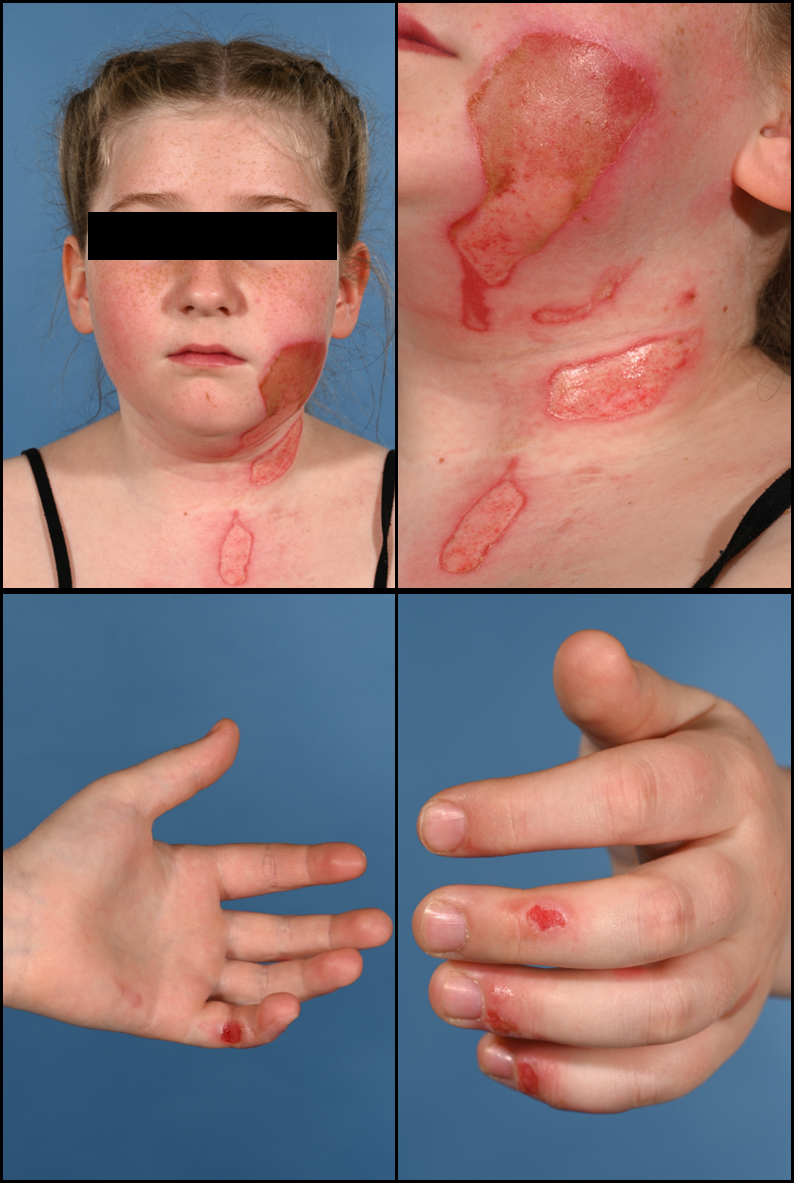

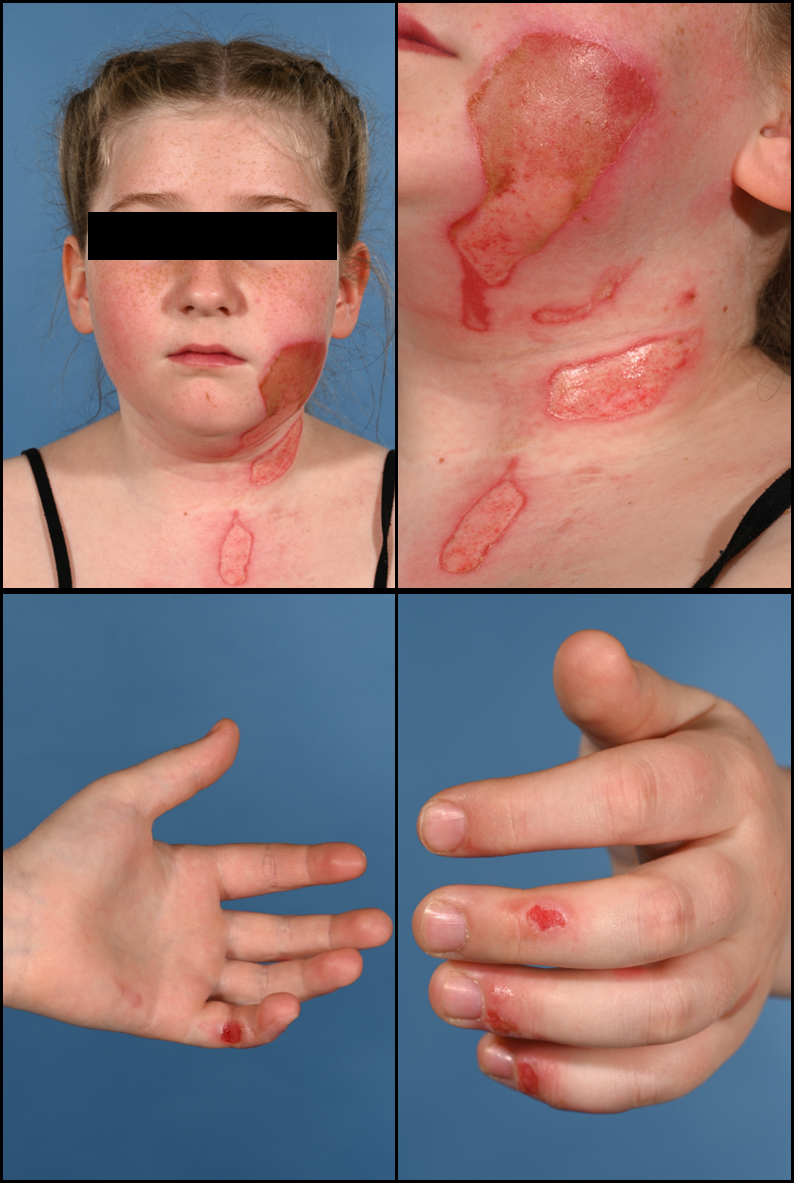

Supplement: Supplementary file 2 [file mmc2.docx]
